# 221. Evaluation of Rates of Culture Positive Blood Stream Pathogens Prior to and During the SARS-CoV-2 Pandemic: A Multicenter Evaluation

**DOI:** 10.1093/ofid/ofab466.423

**Published:** 2021-12-04

**Authors:** Laura A Puzniak, Karri A Bauer, Kalvin Yu, Pamela Moise, Vikas Gupta

**Affiliations:** 1 Merck & Co., Inc., Kenilworth, New Jersey; 2 Merck & Co, Inc, Kenilworth, New Jersey; 3 Becton, Dickinson and Company, Franklin Lakes, New Jersey; 4 Merck Research Labs, Merck & Co., Inc., Kenilworth, New Jersey

## Abstract

**Background:**

Bacterial co-infections or super-infections are well-characterized complications of viral infections, further increasing morbidity and mortality of global viral pandemics. We evaluated trends in the incidence of culture positive gram-negative (GN), gram-positive (GP), and fungal/yeast pathogens from a blood source in hospitalized patients at US hospitals before and during the SARS-CoV-2 pandemic.

Table: Incidence and rate of blood pathogens in the pre and post SARS-CoV-2 period.

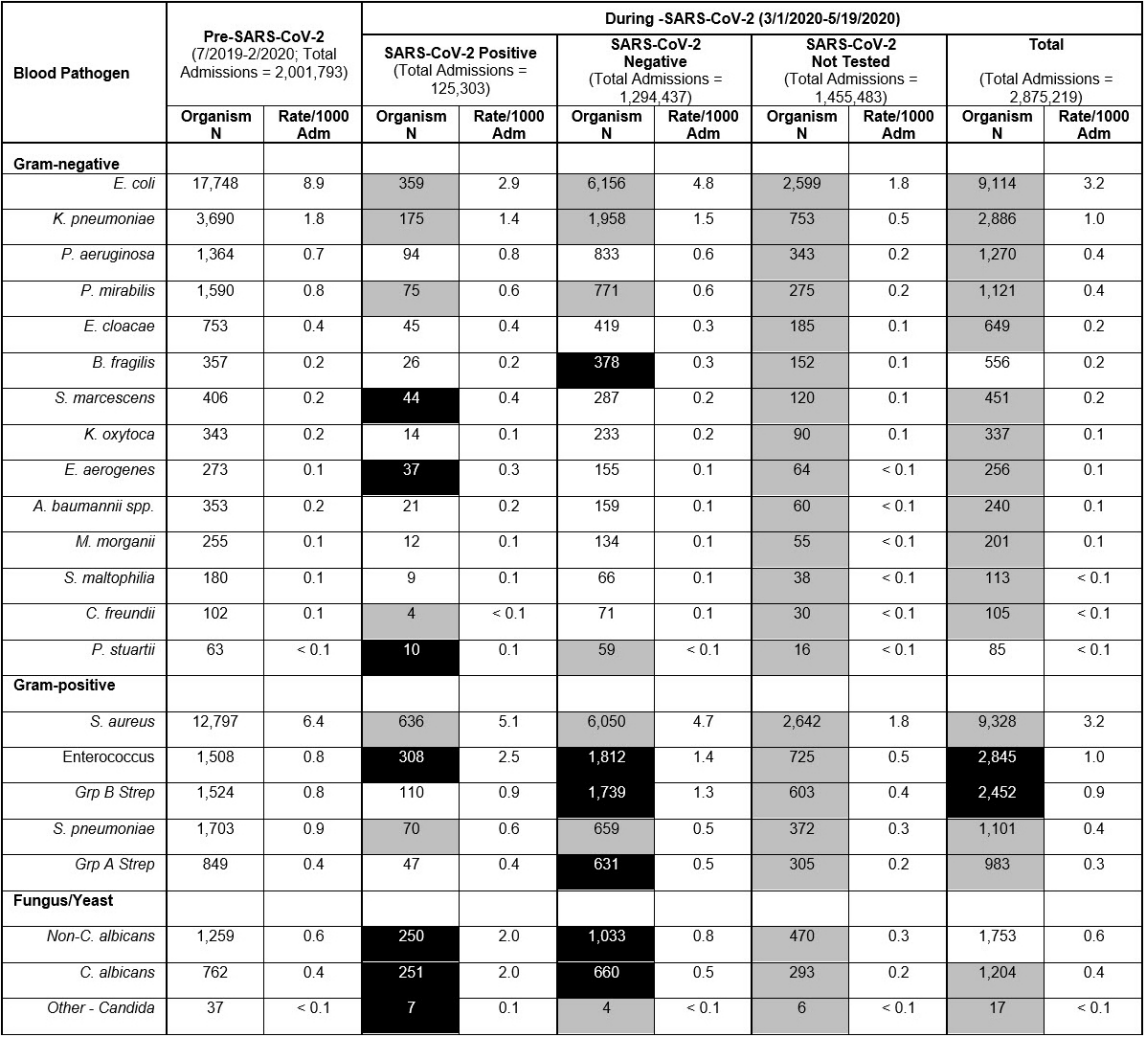

Gray indicates significantly lower rate compared to pre-pandemic time period, black indicates significantly higher rates compared to pre-pandemic.

Methods: This was a multi-center, retrospective cohort analysis of all hospitalized patients from 267 US acute care facilities with >1-day inpatient admission between 7/1/19-5/19/21 (BD Insights Research Database [Becton, Dickinson and Company, Franklin Lakes, NJ]). SARS-CoV-2 infection was identified by a positive PCR during or ≤7 days prior to hospitalization. All admissions with a non-contaminant culture positive GN, GP, and fungal/yeast pathogen from a blood source were evaluated prior to and during the SARS-CoV-2 pandemic as rates per 1,000 admissions (p< .05 for significance).

**Results:**

There were 2,001,793 admissions in the pre-SARS-CoV-2 period (7/2019-2/2020) and 2,875,219 admissions during the SARS-CoV-2 pandemic. Incidence of GN/GP blood stream pathogens was significantly higher prior to the SARS-CoV-2 pandemic than during the pandemic. Higher rates of blood stream pathogens occurred in those who were tested for SARS-CoV-2, but all non-tested patients had significantly lower rates than pre-pandemic. Rates of *Candida spp.*, Enterococcus spp., *Serratia marcescens*, and *Enterobacter cloacae* were higher in SARS-CoV-2 positive patients compared to pre-pandemic patients. Compared to the prior pandemic period, the incidence of *B. fragilis*, *Streptococcus*, Enterococcus and *Candida* were higher among those tested for SARS-CoV-2 but were negative.

**Conclusion:**

In general, rates of positive blood cultures for bacterial pathogens were either lower or similar during the SARS-CoV-2 period compared to the pre-SARS-CoV-2 pandemic period. The patients that were tested for SARS-CoV-2 but were positive who had higher rates of infection than prior may indicate the similarity in viral and bacterial clinical presentation. Further evaluation of higher rates of Enterococcus and Candida in the pandemic period are warranted.

**Disclosures:**

**Laura A. Puzniak, PhD**, **Merck & Co., Inc.** (Employee) **Karri A. Bauer, PharmD**, **Merck & Co., Inc.** (Employee, Shareholder) **Kalvin Yu, MD**, **BD** (Employee) **Pamela Moise, PharmD**, **Merck** (Employee) **Vikas Gupta, PharmD, BCPS**, **Becton, Dickinson and Company** (Employee, Shareholder)

